# Fraction of nitrous oxide production in nitrification and its effect on total soil emission: A meta-analysis and global-scale sensitivity analysis using a process-based model

**DOI:** 10.1371/journal.pone.0219159

**Published:** 2019-07-10

**Authors:** Motoko Inatomi, Tomohiro Hajima, Akihiko Ito

**Affiliations:** 1 Research Center for Agricultural Information Technology, NARO, Tsukuba, Japan; 2 Japan Agency for Marine–Earth Science and Technology, Yokohama, Japan; 3 National Institute for Environmental Studies, Tsukuba, Japan; Chinese Academy of Sciences, CHINA

## Abstract

Nitrification in terrestrial soils is one of the major processes of emission of nitrous oxide (N_2_O), a potent greenhouse gas and stratospheric-ozone-depleting substance. We assessed the fraction of N_2_O emission associated with nitrification in soil through a meta-analysis and sensitivity analysis using a process-based model. We corrected observational values of gross nitrification and associated N_2_O emission rates from 71 records for various soils in the world spanning from 0.006% to 29.5%. We obtained a median value of 0.14%, and then assessed how the nitrification-associated N_2_O emission fraction has been considered in terrestrial nitrogen cycle models. Using a process-based biogeochemical model, we conducted a series of sensitivity analyses for the effects of different values of nitrification-associated N_2_O emission fraction on soil N_2_O emission. Using an empirical relationship between soil pH and nitrification-associated N_2_O emission fraction, the model well simulated global emission patterns (global total in the 2000s, 16.8 Tg N_2_O yr^–1^). Differences in the nitrification-associated N_2_O emission fraction caused differences in total N_2_O emission of as much as 2.5 Tg N_2_O yr^–1^. Therefore, to obtain reliable estimation of soil N_2_O emission for nitrogen and climate management, it is important to constrain the parameterization in models by ensuring extensive and accurate observations.

## Introduction

Nitrous oxide (N_2_O) is the third important long-lived greenhouse gas next to carbon dioxide (CO_2_) and methane (CH_4_) [1] and is the most important substance depleting stratospheric ozone [2]. To reach the overarching mitigation targets of the Paris Agreement [3] we need to suppress the growth of atmospheric N_2_O concentration, to which anthropogenic emissions contribute at a level comparable to that from natural sources [4, 5]. Also, assessment and regulation of N_2_O emission contribute to management of nitrogen cycle, which is closely related to many issues of human sustainability, such as food production and sanitation [6]. Nevertheless, there remain serious uncertainties in our understanding and predictability of N_2_O dynamics.

Terrestrial soils—both natural and agricultural—are a prevailing source of N_2_O in the atmosphere [7, 8], but spatial heterogeneity and temporal variability of the N_2_O flux make it difficult to quantify broad-scale budgets. Most of the N_2_O released from the soil surface is produced by two separate microbial processes, nitrification and denitrification, which differ in terms of active microbes, substrates, and environmental responsiveness [9, 10]. There are still serious knowledge gaps and difficulties in using models to predict soil N_2_O emissions in a quantitative manner.

Nitrification by ammonia oxidizers is the primary process of N_2_O production in oxic (aerobic) soils and is thought to be more ubiquitous than denitrification, which occurs in anaerobic wet soils. Recent studies have revealed the contributions of different soil microbes, such as ammonia-oxidizing archaea and bacteria, to nitrification [11, 12]. In nitrification, most of the oxidized ammonia is turned into nitrate (NO_3_^–^) via nitrite (NO_2_^–^), and a certain (usually small) fraction of nitrogen is released as N_2_O. The fraction of nitrification-associated N_2_O emission (fN_2_O_nit_) and its regulation mechanism are important but barely understood. Although fN_2_O_nit_ is critically important to predict soil N_2_O emission, a few studies have investigated the responses of fN_2_O_nit_ and the corresponding nitric oxide (NO) emission fraction to soil temperature and moisture conditions [13, 14]. However, observational data and knowledge are still insufficient to evaluate broad-scale emissions, including from a variety of soils. Farquharson (2016) [15] conducted a systematic analysis of fN_2_O_nit_ from agricultural soils in Australia. He found that 0.03% to 1% of nitrogen is released as N_2_O associated with nitrification in soils and found no strong relationship with environmental factors such as soil moisture. For a broad range of natural soils, and in other regions, we have found no systematic analysis on fN_2_O_nit_.

**Fig 1:**
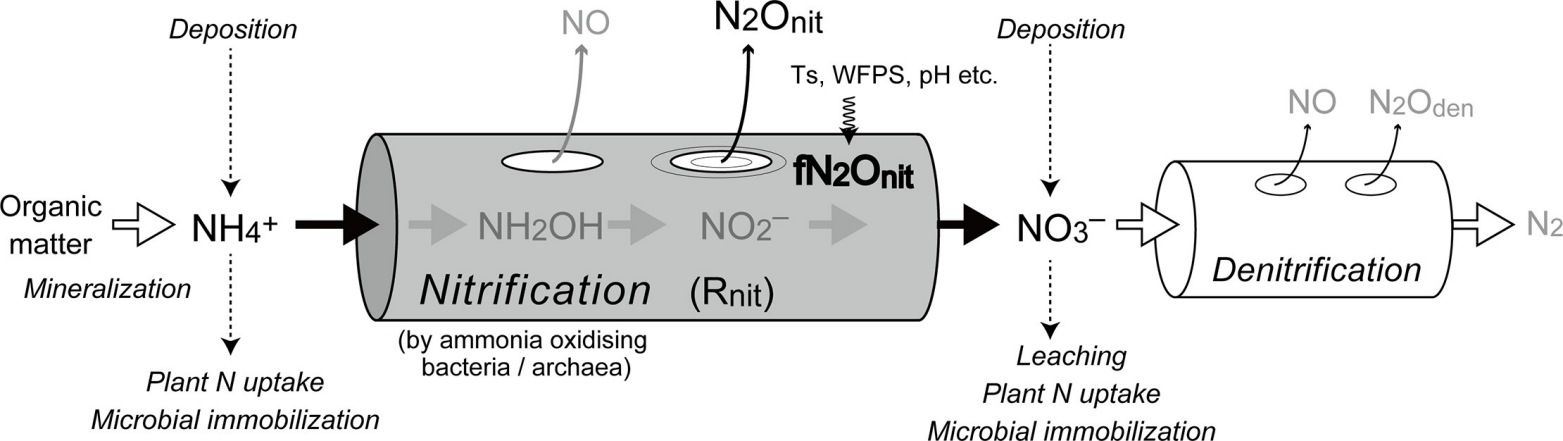
Conceptual diagram of nitrification and the “holes-in-a-pipe” concept. Ts, soil temperature; WFPS, water-filled pore space.

fN_2_O_nit_ should be an important parameter in biogeochemical models that aim to simulate nitrogen cycles and predict N_2_O emissions from land. Many terrestrial nitrogen cycle and N_2_O emission models have been developed, from simple box-flow models (e.g., the Terrestrial Ecosystem Model [16]) to more mechanistic ones (e.g., the Denitrification Decomposition [DNDC] model [17]). In these models, fN_2_O_nit_ or a similar parameter (i.e., the total N_2_O and NO emission fraction) has been determined in a simplified empirical manner. In the well-recognized “hole in the pipe” or “leaky pipe” concept of soil nitrogenous gas emission [18, 19], fN_2_O_nit_ represents the size of the N_2_O hole in the nitrification pipe (Fig 1). Note that the nitrification rate (as defined by pipe diameter and flow velocity) varies also with the environmental conditions, so it is possible to regard fN_2_O_nit_ as a constant or as an independent variable that changes with environmental condition. In the latter case, empirical parameterizations have been adopted to determine the N_2_O emission fraction, using a limited amount of observational data. As a consequence, these emissions could have a considerable range of bias and error due to the uncertainty of fN_2_O_nit_ values.

Here, we focused on fN_2_O_nit_ from the perspective of the global N_2_O budget, aiming at better predictability of emission by biogeochemical models. Our focal research questions are as follows. (1) What is the feasible range of fN_2_O_nit_ in the terrestrial ecosystems? (2) How does fN_2_O_nit_ vary in the field in response to environmental conditions? (3) Can we attain a better parameterization of fN_2_O_nit_, applicable to global scale, on the basis of present data? First, to clarify the range of variability and the broad-scale trends in these values, we conducted a meta-analysis of the observed values of fN_2_O_nit_ for both natural and agricultural soils. Second, we surveyed how fN_2_O_nit_ was included in current terrestrial N_2_O estimation models. Third, we used the results of the meta-analysis and model survey to conduct a series of sensitivity analyses of fN_2_O_nit_ and N_2_O emissions by using our biogeochemical model. Finally, we discuss how we can reduce the range of estimation uncertainty in both experimental and modeling studies. Note that this study focuses on nitrification as the first step and that other important processes such as denitrification, nitrifier denitrification, and abiotic production [20] are not explicitly addressed here. We intend to address these other processes in a forthcoming study using a similar approach.

## Methods

### Meta-analysis

#### Overview

A meta-analysis was conducted to reveal the range, frequency, and tendencies of soil fN_2_O_nit_ reported in the literature. The results were reported following the Preferred Reporting Items for Systematic Review and Meta-Analyses protocols (PRISMA) [21] protocol (S1 Table). To obtain information on the general properties of the parameter, we gathered observational values reported from a wide range of studies. By using Web of Science (Thomson Reuters, New York, NY, USA) and Google Scholar (Alphabet, Mountain View, CA, USA), we searched papers and reports that included data on nitrogenous gas exchange and soil biogeochemistry. We used combinations of three terms (each from #1 to #3) in S2 Table to search candidate papers; for example, “nitrous oxide flux” and “nitrification rate” and “soil surface”. No date and time limitations were applied to harvest from the maximum extent of the literature. Also, we examined the reference lists in each paper to find additional literature that did not appear in the web searches.

#### Study selection

We carefully selected source data of fN_2_O_nit_ for the meta-analysis, particularly taking into account the consistency between N_2_O emission and nitrification rates. First, we removed papers that addressed non-soil N_2_O emissions (i.e., from ponds, landfills, animal slurry, etc.). For quantitative consistency, we selected papers including data on gross nitrification (i.e., NH_4_^+^ consumption) and associated N_2_O emission. Therefore, several papers that reported only net nitrification (i.e., NO_3_^–^ production) rates, potential emission rates, and data under oxygen-free condition, were carefully removed from the meta-analysis. Also, we focused on daily or longer phenomena, so that rates of N_2_O emission from the soil surface to the atmosphere could be adequately approximated to N_2_O production rates within the soil (i.e., the vertical diffusion time lag was negligible). As a result, papers reporting only instantaneous (i.e., for seconds to minutes) measurements were excluded; in general, these instantaneous measurement data show extremely wide ranges of variability, making a robust analysis difficult. Finally, we selected the source papers by measurement method used in each study, because several methods could give biased values under certain conditions (e.g., DMPP inhibition slows greatly at >25°C [22]). We confirmed that the method-based selection had a small impact on the analysis results. The paper selection was made by two authors and discrepancies were resolved by discussion.

#### Data extraction

Data on fN_2_O_nit_ values and associated properties were extracted: soil pH, solvent of pH, soil temperature, soil moisture content with units, soil texture, clay / silt / sand composition, latitude, longitude, land-cover type, and soil-type classification. Few papers provide values of fN_2_O_nit_ directly, and therefore, if applicable, we calculated the values from gross nitrification and associated N_2_O emission rates measured under the same condition.

#### Data analyses

A statistical software R [23] was used to calculate the statistical metrics for the records: i.e., mean, median, maximum, minimum, standard deviation [σ], kurtosis, skewness, and quartiles. A few extreme values can, in most cases harmfully, affect the results of statistical metrics. To assess the influences of outliers, these statistical metrics were also calculated after removing top and bottom outliers (10% from all the records). Note that we used both datasets with and without outlier values in the following analyses and model simulations. Furthermore, to reduce the size effect of different sample numbers (i.e., weights) among papers, these metrics were also calculated using the mean values for each paper.

### Parameterization of fN_2_O_nit_ in other models

**Fig 2:**
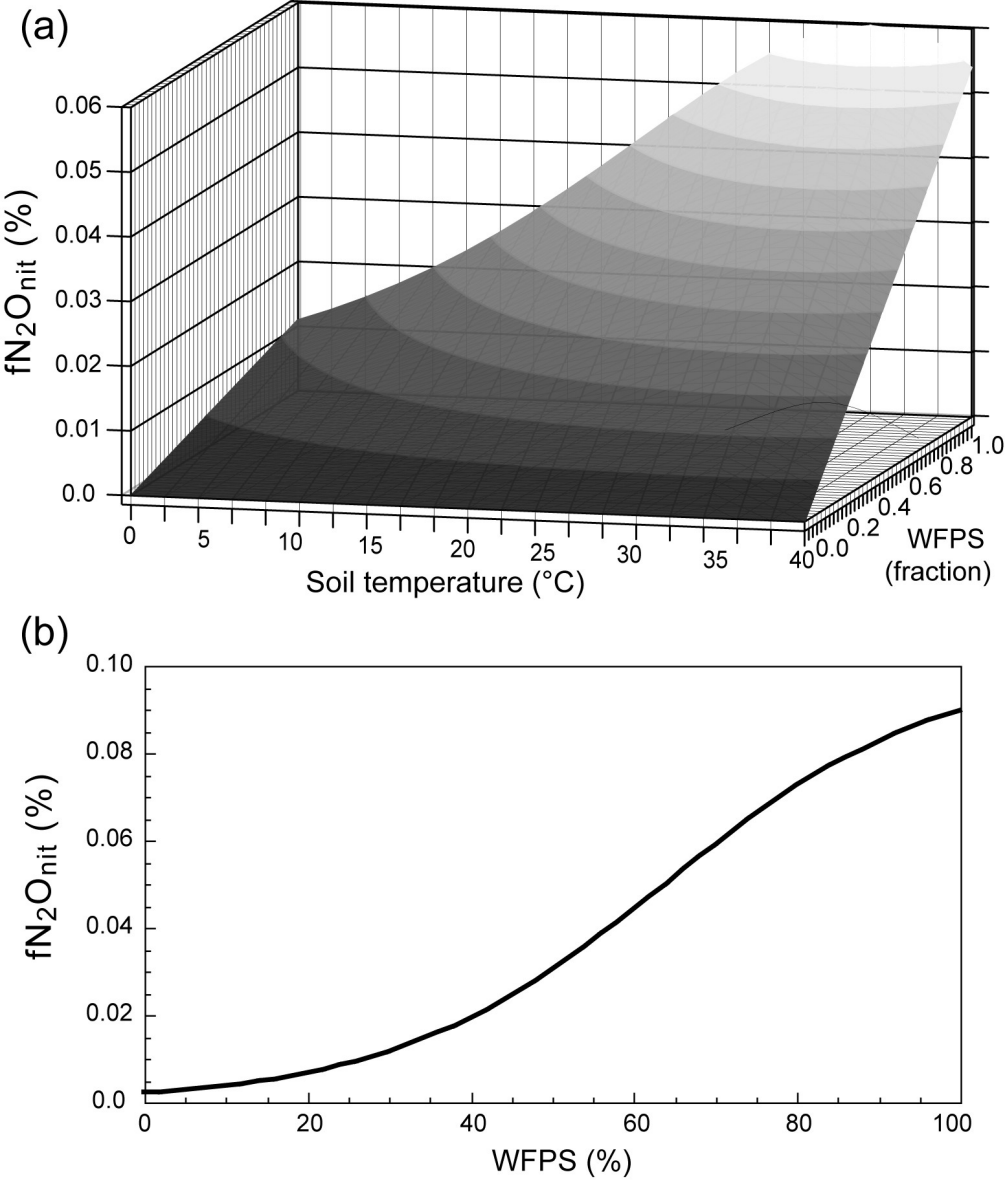
Relationship between the fraction of nitrification-associated N_2_O emission (fN_2_O_nit_) assumed in the models. (a) DNDC and (b) DLEM. WFPS, water-filled pore space.

We then assessed how fN_2_O_nit_ has been parameterized in other terrestrial nitrogen models and evaluated the influence on N_2_O emission estimation. According to a review by Frolking et al. (1998) [24], former models have adopted different constant fN_2_O_nit_ values, namely 0.5% in the ExpertN model and 2% in the CENTURY model. These values were examined in the sensitivity simulations mentioned below. In later models, fN_2_O_nit_ was parameterized as a function of environmental conditions in different manners. The modified DNDC model [25] (their Table 3, Eq 8) adopted the following parameterization:

$$fN_{2}O_{nit}=0.06∙Ft*WFPS,$$ (1)

$$Ft={\left[(60-Ts)/25.78\right]}^{3.503}exp[3.503(Ts-34.22)/25.78],$$ (2)

where Ft is a scholar function of soil temperature (Ts,°C) and WFPS is water-filled pore space (fraction). This parameterization gives a peak value, about 0.06%, at about 35°C under saturated soil water conditions (Fig 2A). The Dynamic Land Ecosystem Model [26] (DLEM) parameterizes fN_2_O_nit_ as a function of WFPS (%):

$$fN_{2}O_{nit}=0.1∙10^{0.026∙WFPS-1.66}/(1+10^{0.026∙WFPS-1.66}),$$ (3)

In this parameterization, the fN_2_O_nit_ value increases with increasing soil water content (Fig 2B) and does not exceed 0.1%. The Community Land Model [27] and O-CN model [28] adopted a version of the DNDC parameterization. In the recent paper on N_2_O model intercomparison [29], an elaborate table summarizes how nitrification and N_2_O emission are formulated in contemporary land nitrogen models.

### Description of the N_2_O simulation model

To assess the range of estimations associated with variations in fN_2_O_nit_, we conducted a series of simulations using a process-based model, namely, Vegetation Integrative SImulator for Trace gases (VISIT [30, 31]). This model was selected, because it has an intermediate complexity among the global N_2_O models and gave moderate results in the model intercomparison project [29]. Also, the model was used for a regional evaluation of soil N_2_O emission in East Asia, one of the highly human-influenced regions, demonstrating the credibility for broad-scale applications [32]. Briefly, the model consists of water, carbon, and nitrogen cycling schemes for terrestrial ecosystems and is aimed at assessing atmosphere–ecosystem exchange of greenhouse gases and trace gases. The nitrogen cycle is fully included, from inputs (atmospheric deposition, biological fixation, and fertilizer input) to outputs (leaching, ammonia volatilization, and nitrogenous gas emissions through nitrification and denitrification). Intra-ecosystem dynamics of nitrogen among plant, soil, and microbe is simulated in an explicit manner. The model was validated by comparing various biogeochemical aspects with observational data [33–35]. In the VISIT model, fN_2_O_nit_ is assumed to be a universal, constant value (1% of gross nitrification).

A brief description of the method used to estimate fN_2_O_nit_ is given below. In the VISIT model, soil N_2_O production through the nitrogen cycle is conceptualized by using the “hole in the pipe” scheme (see Fig 1). Gross nitrification (R_nit_) and associated N_2_O emission (N_2_O_nit_) are related as follows:

$$N_{2}O_{nit}=fN_{2}O_{nit}∙R_{nit},$$ (4)

Nitrification rate and its environmental dependencies were derived from the NGAS scheme developed by Parton et al. (1996) [36], as follows:

$$R_{nit}=p_{WFPS-nit}∙p_{pH}∙p_{Ts}(K+F_{max}∙NH_{4}^{+})/fN_{2}O_{nit},$$ (5)

where *p*_WFPS-nit_, *p*_pH_, and *p*_Ts_ denote the environmental scalar functions derived from observations (see ref. [36]) of water-filled pore space (WFPS), soil pH, and soil temperature (Ts), respectively. K denotes the coefficient of N turnover, taking values from 3.5 of natural soils to 12.0 of agricultural soils. F_max_ is the maximum nitrification-associated gas flux coefficient and NH_4_^+^ is the soil ammonium content. WFPS and NH_4_^+^ were simulated by VISIT and so varies temporally and spatially.

Global simulations by using the VISIT model were conducted with the common protocol and initial and boundary conditions. Namely, they were conducted at a spatial resolution of 0.5° x 0.5° for latitude and longitude, during the period from January 1901 to December 2016. Historical climate data from CRU TS3.25 [37] (temperature, precipitation, vapor pressure, and cloudiness) and land-use data [38] were used to drive the model. Historical changes in atmospheric nitrogen deposition were derived from Galloway et al. (2004) [39], and in croplands, input of nitrogen fertilizer was determined on a country-basis by using FAOSTAT (http://www.fao.org/faostat). The amount of national fertilizer use was divided by total cropland area and allocated to each grid cell. For each grid, a spin-up calculation was conducted for 300 to 2000 years, depending on case, under stationary conditions until a stable-state carbon budget was reached, before starting the historical experiment.

### Sensitivity simulations

All sensitivity simulations were conducted by VISIT using the common forcing dataset and protocols; only the fN_2_O_nit_ value was changed. First, to simply assess the sensitivity of N_2_O emission estimation to fN_2_O_nit_, we halved (i.e., 0.5%) and doubled (2%) the parameter value (originally 1% in VISIT) and compared the results. We then changed the fN_2_O_nit_ values to those obtained by the meta-analysis mentioned above, that is, mean and median values for all data and several subsets. In these simulations, constant fN_2_O_nit_ values were applied to all grids. Second, the fN_2_O_nit_ value was replaced by those of DNDC and DLEM described above, using the same temperature and moisture conditions. Third, finally, we derived an empirical relationship between soil pH and fN_2_O_nit_ value from the meta-analysis data. Such a relationship was shown in laboratory studies [40] and previous meta-analysis [15], but has not been examined by models at the global scale. Here, we used the global soil pH map (S1 Fig) produced by the Global Soil Data Task of the International Geosphere-Biosphere Programme [41].

## Results and discussion

**Fig 3:**
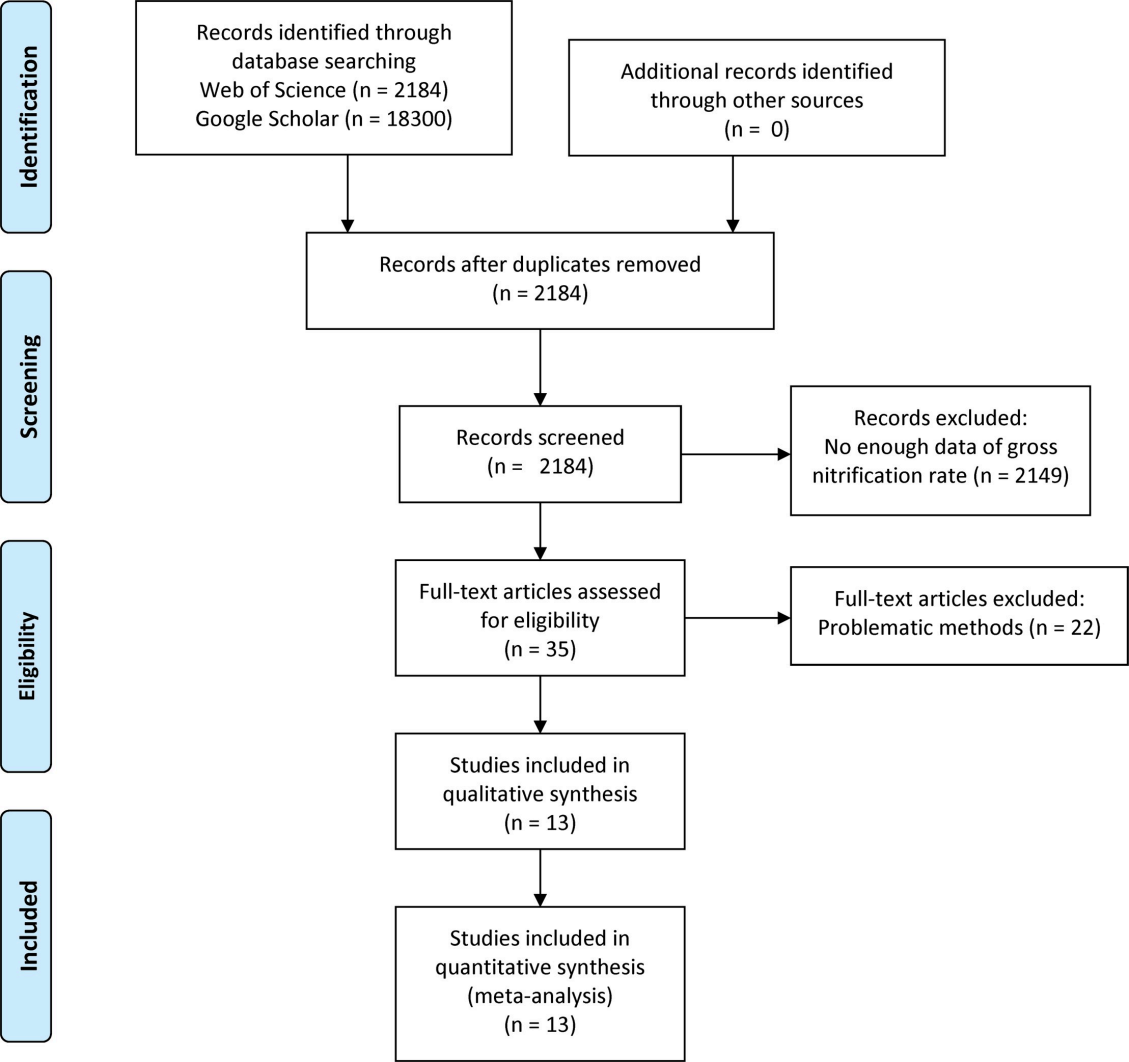
PRISMA flow diagram. PRISMA, Preferred Reporting Items for Systematic Reviews and Meta-Analysis.

We obtained 71 records from 13 studies in the published literature (Table 1; Fig 3), covering a wide range of different ecosystems and soil texture types; see S3 Table for the data extracted. Although no date and time limitation were applied, the data were obtained from 1985 to 2013. Although a large number of papers addressed the nitrification and N_2_O emission (2184 papers), we found that only a small number of papers (35 papers) contain the data on gross nitrification rate for a sufficiently long period. Other 2149 papers were rejected, although they contained partial data on nitrification-associated N_2_O emission. Furthermore, many of the measurements (22 out of 35) were made using problematic methods or conditions and so removed. As a result of data selection, measurements in the literature used in this study were made mainly by using two methods of soil incubation: the inhibitor (C_2_H_2_, N-serve, and NaClO_3_) treatment and the stable carbon isotope (^15^N labelling) method [10, 42]. Most of the measurements were conducted in the laboratory: only one study was done in the field. The record number is not so abundant, but the dataset covers a wide variety of ecosystems and soils such as forest, grassland, and cropland. Therefore, we used this dataset for following analyses and model assessments.

**Table 1:** List derived from literature search of methods to measure nitrification-associated N_2_O flux.

References	Method	fN_2_Onit	Note
		mean (max ~ min), %	
Ambus (2005) ref. [43]	^15^N labelling	0.046 (0.046 ~ 0.046)	Land cover (sward), soil type (Typic Hapludult)
Bateman and Baggs (2005) ref. [14]	^15^N labelling, C_2_H_2_ inhibition	0.011 (0.006 ~ 0.014)	Land cover (agriculture), soil type (Cambion)
Carter (2007) ref. [44]	C_2_H_2_ inhibition, field measurement (^15^N)	0.020 (0.01 ~ 0.029)	Land cover (sward)
Garrido *et al*. (2002) ref. [45]	C_2_H_2_ inhibition	0.30 (0.028 ~ 0.48)	Land cover (agriculture), soil type (HypercalcareousRendosol, RedoxicLuvisol, PachicCalcisol, Neoluvisol)
Khalil *et al*. (2004) ref. [46]	^15^N labelling	0.77 (0 ~ 1.57)	Land cover (agriculture), soil type (Orthic Luvisol)
Klemedtsson *et al*. (1988) ref. [47]	C_2_H_2_ inhibition	-0.49 (-9.62 ~ 7.5)	Land cover (arable land)
Maag and Vinther (1996) ref. [48]	C_2_H_2_ inhibition	0.36 (0.28 ~ 0.48)	
Martikainen (1985) ref. [40]	C_2_H_2_ inhibition, N-serve inhibition	28.3 (27.0 ~ 29.4)	Land cover (forest)
Mathieu *et al*. (2006) ref. [49]	^15^N labelling	1.23 (0.13 ~ 2.32)	Land cover (agriculture), soil type (Gleyie luvisol)
Mørkved *et al*. (2007) ref. [50]	^15^N labelling, C_2_H_2_ inhibition	0.79 (0.018 ~ 7.62)	Land cover (meadow, agriculture), soil type (sapric histosol, Stagnic Albeluvisol)
Mørkved *et al*. (2006) ref. [51]	^15^N labelling, C_2_H_2_ inhibition	27 (27 ~ 27)	Land cover (agriculture), soil type (mollic gleysol)
Tortoso and Hutchinson (1990) ref. [52]	N-serve inhibition, NaClO_3_ inhibition	0.068 (0.068 ~ 0.068)	Land cover (agriculture)
Zhu *et al*. (2013) ref. [20]	^15^N labelling, ^18^O, C_2_H_2_ inhibition	2.35 (0 ~ 8.3)	Land cover (agriculture), soil type (Fine-silty mixed, nonacid, thermic Typic Xerorthent)

N-Serve, 2-Chloro-6-(trichloromethyl)-pyridine

**Fig 4:**
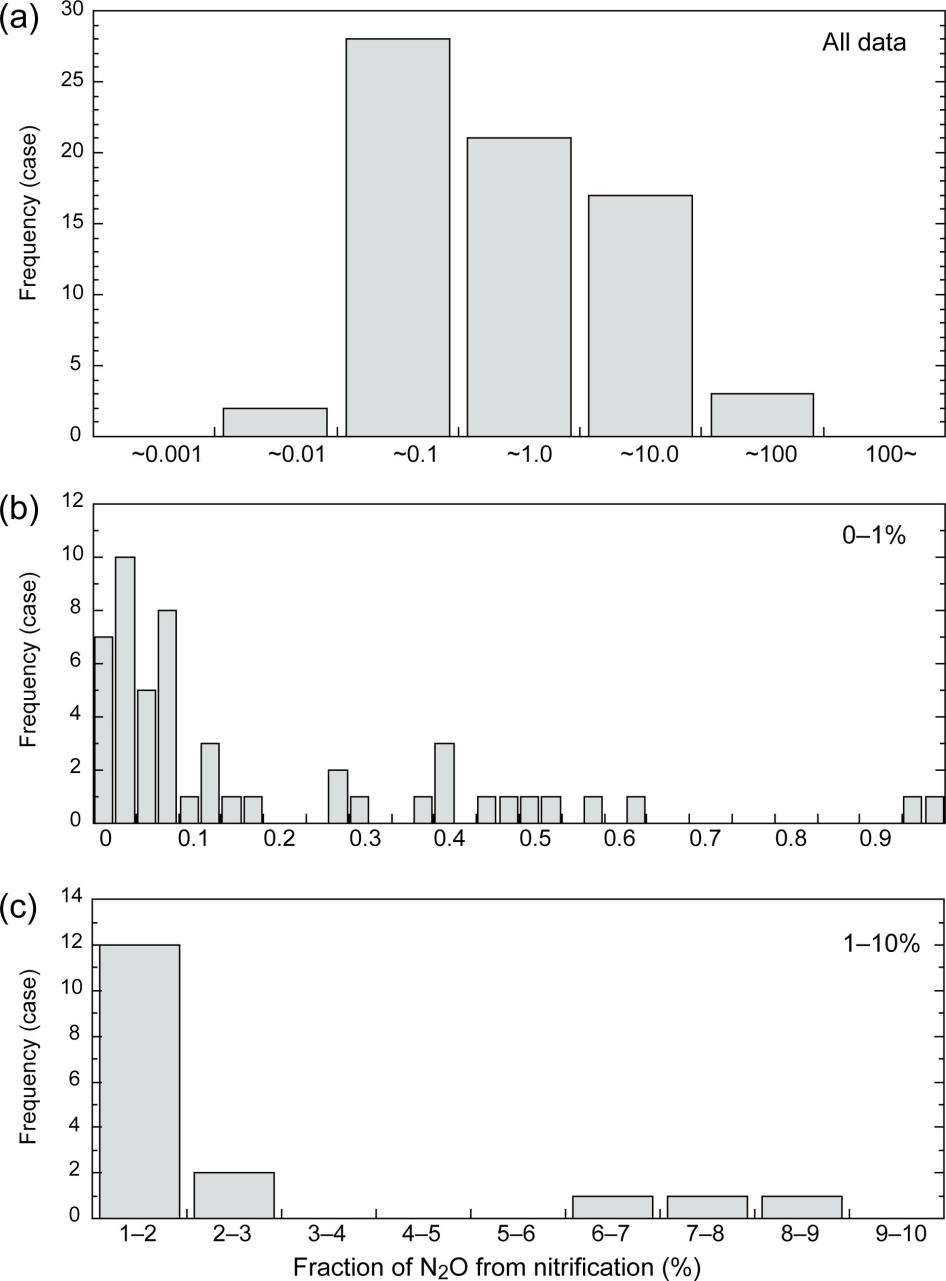
Histogram of the N_2_O emission associated with nitrification, obtained by a meta-analysis of 71 observations. (a) All data, (b) data of 0–1%, and (c) data of 1–10%.

The observational fN_2_O_nit_ values (n = 71) were distributed widely from 0.006% to 29.4%, with a mean of 1.92% and a median of 0.19% (first column of Table 2). The high mean value was likely attributable to the presence of a few anomalous values in the dataset. A histogram of the observed fN_2_O_nit_ values (Fig 4) showed a concentrated and skewed distribution with a clear peak around the median value. Additionally, with removal of 10% outliers (top 4 and bottom 3 records, second column of Table 2) the mean (0.43%) and median (0.14%) values became lower. Notably, standard deviation narrowed greatly after the removal of outliers, and both the maximum value and minimum value were obtained when the C_2_H_2_ inhibition method was used. When publication-based data (i.e., the means of the values reported in each paper) were used, the mean and median value became higher (5.17% and 0.57%, respectively; third column of Table 2).

**Table 2:** Summary of statistics on measured nitrification N_2_O emission ratio.

	All data	Excluding outlier	Aggregated by paper
Sample no.	71	64	12
Mean (%)	1.922	0.426	5.167
Standard deviation (%)	5.702	0.526	10.526
Kurtosis (-)	15.255	1.191	0.498
Skewness (-)	4.006	1.369	1.549
Maximum (%)	29.445	2.320	28.234
75% quartile (%)	1.084	0.605	1.703
Median (%)	0.192	0.139	0.573
25% quartile (%)	0.053	0.048	0.063
Minimum (%)	0.006	0.006	0.011

In comparison with the study of Australian agricultural soils by Farquharson (2016), typical values of fN_2_O_nit_ obtained by our meta-analysis seem comparable. That study found that fN_2_O_nit_ values varied from 0.03% to 1%, with a typical value of 0.2%, that falls between the mean and median values obtained in the present study for all data. When removing outlier values, our results of mean and median became even closer to the result of Farquharson (2016). Namely, we examined anomalous values removed from the present meta-analysis, such as zero to negative and extremely high, 100% values. Several these values were obtained by Ambus (1998) [53] in the only study that conducted C_2_H_2_ treatment in the field; this probably led to a larger fluctuation in values than in the laboratory studies. Negative values of fN_2_O_nit_ are attributable to net N_2_O uptakes, which are sometimes observed but are usually small [54]. The fN_2_O_nit_ values used by emission models assuming a constant N_2_O emission fraction (0.5% to 2.0%) fell within the range of observed values. For example, the constant fN_2_O_nit_ value used in the original VISIT model, 1%, did not differ significantly from the mean value of the observed dataset (Student’s t-test, *t* = 1.36, *p* = 0.177). However, it should be noted that the median observed value (0.19%) was much lower than the model-assumed value.

**Fig 5:**
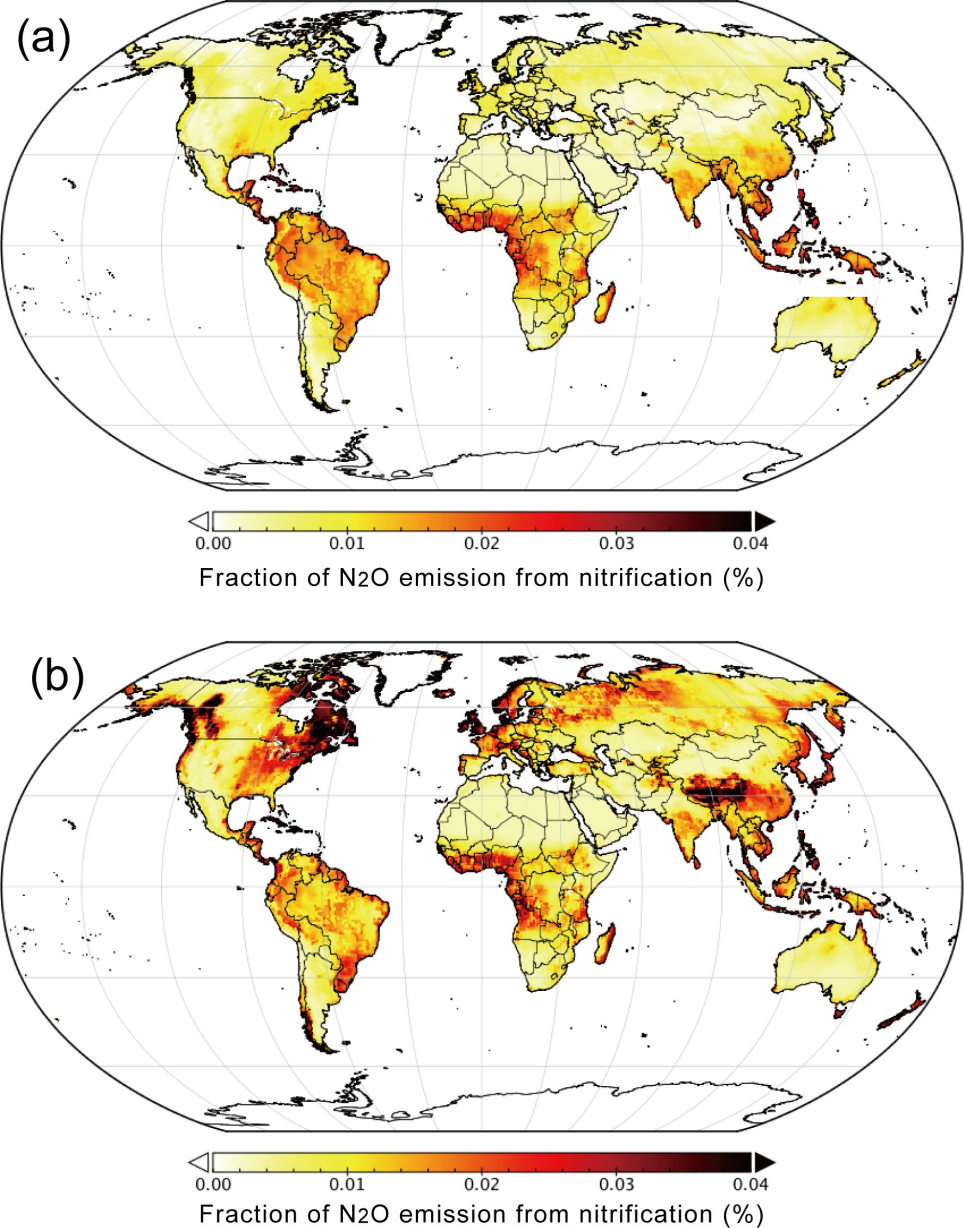
Distributions of estimated fraction of nitrification-associated N_2_O emission, fN_2_O_nit_. (a) DNDC and (b) DLEM parameterizations.

The fN_2_O_nit_ values used in the DNDC and DLEM models including the environmental variability of the N_2_O emission fraction were generally lower than those used in the models assuming constant values. As shown in Fig 2, these models assume the maximum fN_2_O_nit_ values of 0.06 to 0.1%. However, these fN_2_O_nit_ values were still within the range of observed values. We examined the spatial distribution of mean fN_2_O_nit_ values estimated by the DNDC (Fig 5A) and DLEM (Fig 5B). When the DNDC parameterization was used, higher fN_2_O_nit_ values were estimated mainly in the humid tropics. In contrast, when the DLEM parameterization was used, higher fN_2_O_nit_ values were obtained in humid temperate to boreal regions such as Europe, eastern and western North America, and the Tibetan Plateau. Note again that a common soil temperature and moisture (after the VISIT simulation) were used in this comparison, and the differences among the results were caused exclusively by differences in the fN_2_O_nit_ parameterizations. The difference of fN_2_O_nit_ indicated here may account for a part of outcomes of the N_2_O model intercomparison project [29]. The project showed that the existing models differ in global soil N_2_O emission by about 20%, and our study implies that fN_2_O_nit_ is one of the key parameters to reduce the estimation uncertainty.

### Sensitivity of N_2_O flux to fN_2_O_nit_

**Fig 6:**
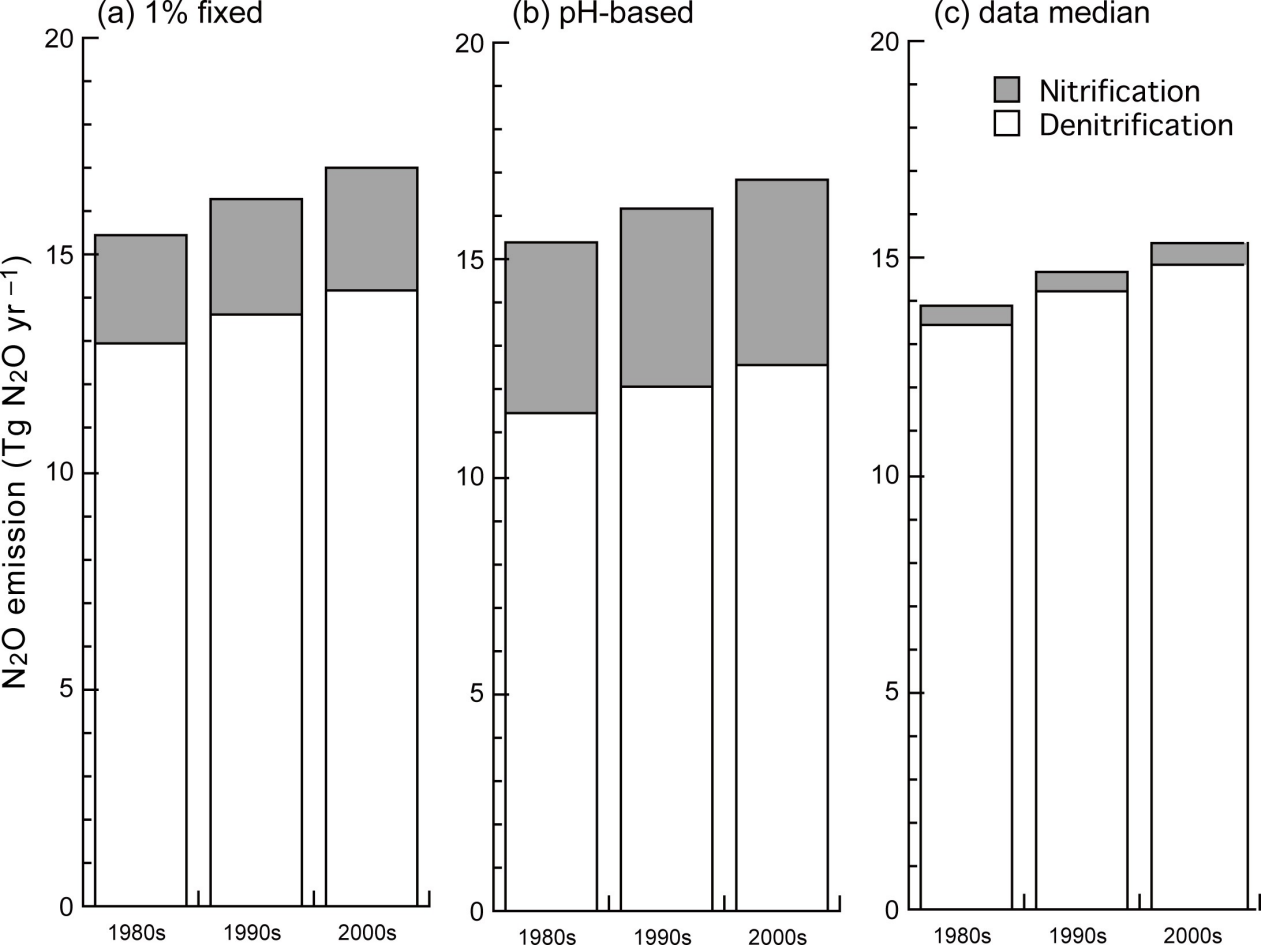
Sensitivity analysis of global N_2_O emission to the fraction of nitrification-associated N_2_O emission, fN_2_O_nit_. (a) Fixed 1%, (b) soil pH-based parameterization, and (c) median of meta-analysis records (0.139%, outliers removed). Each N_2_O flux was estimated by using the VISIT model. Decadal mean values for the 1980s, 1990s, and 2000s are shown.

The default VISIT model with a constant fN_2_O_nit_ of 1% estimated total N_2_O emission from terrestrial soils as 15.47 ± 0.52, 16.32 ± 0.98, and 17.03 ± 0.73 Tg N_2_O yr^–1^ (average ± s.d. of interannual variability) in the 1980s, 1990s, and 2000s, respectively (Fig 6). When converted into nitrogen weight (multiplied by 28/44), these values correspond to 9.85 to 10.84 Tg N yr^–1^. These estimates are close to previous estimations: e.g., 11.1 Tg N yr^–1^ by IPCC (2013) [1] and 11.4 Tg N yr^–1^ by Syakila and Kroeze (2011) [55] for emissions from natural vegetation, agriculture, and deposition on land. In the 2000s simulation, 16.5% of total N_2_O emission was from nitrification and 84% was from denitrification. About 32% and 68% of N_2_O emission occurred in agricultural and natural ecosystem soils, respectively. During the simulation period, total N_2_O emission increased from 12.11 Tg N_2_O yr^–1^ in 1901 to 18.60 Tg N_2_O yr^–1^ in 2016 as a result of the increase of fertilizer and deposition inputs. See our resent study [32] on the temporal change and its driver of the regional N_2_O emissions.

The sensitivity analysis using different values of fN_2_O_nit_ (constant) indicated that the simulated total N_2_O emission was sensitive to the assumed emission fraction. When fN_2_O_nit_ = 0.5% was used, the total nitrification-associated N_2_O emission was reduced to 1.59 Tg N_2_O yr^–1^ (–43% in comparison with fN_2_O_nit_ = 1% case) in the 2000s. Because of the smaller nitrogen loss by nitrification in these cases, N_2_O emission from denitrification increased slightly because of the use of excess inorganic nitrogen in the soils. As a result of compensation, total N_2_O emission was only slightly affected (–4.3%) by the halved fN_2_O_nit_ value. When fN_2_O_nit_ = 2% was used, total nitrification-associated N_2_O emission increased to 4.4 Tg N_2_O yr^–1^ (+56.6%). The asymmetric sensitivity of nitrification-associated N_2_O emission to the change in fN_2_O_nit_ value is attributable to alteration of the nitrogen stock in the soils and the non-linear response of N_2_O emission to nitrogen availability [36]. Finally, when using the parameterizations of DNDC and DLEM models, lower total N_2_O emissions were estimated (15.0 Tg N_2_O yr^–1^) with lower contribution of nitrification-associated emission due to the generally low value of fN_2_O_nit_ (data not shown).

When the median fN_2_O_nit_ value of the meta-analysis (0.14%) was used in the VISIT simulation, the total N_2_O emission was estimated as 15.4 Tg N_2_O yr^–1^ in the 2000s; nitrification-associated N_2_O emission was largely reduced to 0.50 Tg N_2_O yr^–1^. In contrast, when the mean value of all data (fN_2_O_nit_ = 1.92%) was used, higher rates of total and nitrification-associated N_2_O emission (17.5 and 4.3 Tg N_2_O yr^–1^) were estimated. Therefore, selection of representative fN_2_O_nit_ value can affect the simulation result by as much as 2.1 Tg N_2_O yr^–1^ at the global scale. When including the difference in model parameterizations, the uncertainty becomes even larger to 2.5 Tg N_2_O yr^–1^.

These results confirmed that the estimated N_2_O emission was sensitive to the assumed fN_2_O_nit_ value, which was poorly constrained in the present models and varied with selection of the metric from the observational data. Although observations implied that the fN_2_O_nit_ can be variable in response to environmental conditions such as temperature and moisture, the scarcity of observational evidence has prevented us to use a standard parameterization and permitted us to assume constant values. Apparently, additional constraints and new parameterizations are required to obtain a reliable N_2_O budget and its flow components. Observational data and insights are accumulating with support of technical developments such as isotopic tracers, but it would take decades to obtain a comprehensive dataset with enough coverage. Next, we made an attempt to develop a new parameterization of fN_2_O_nit_ applicable at the global scale.

### Application of pH-based parameterization

**Fig 7:**
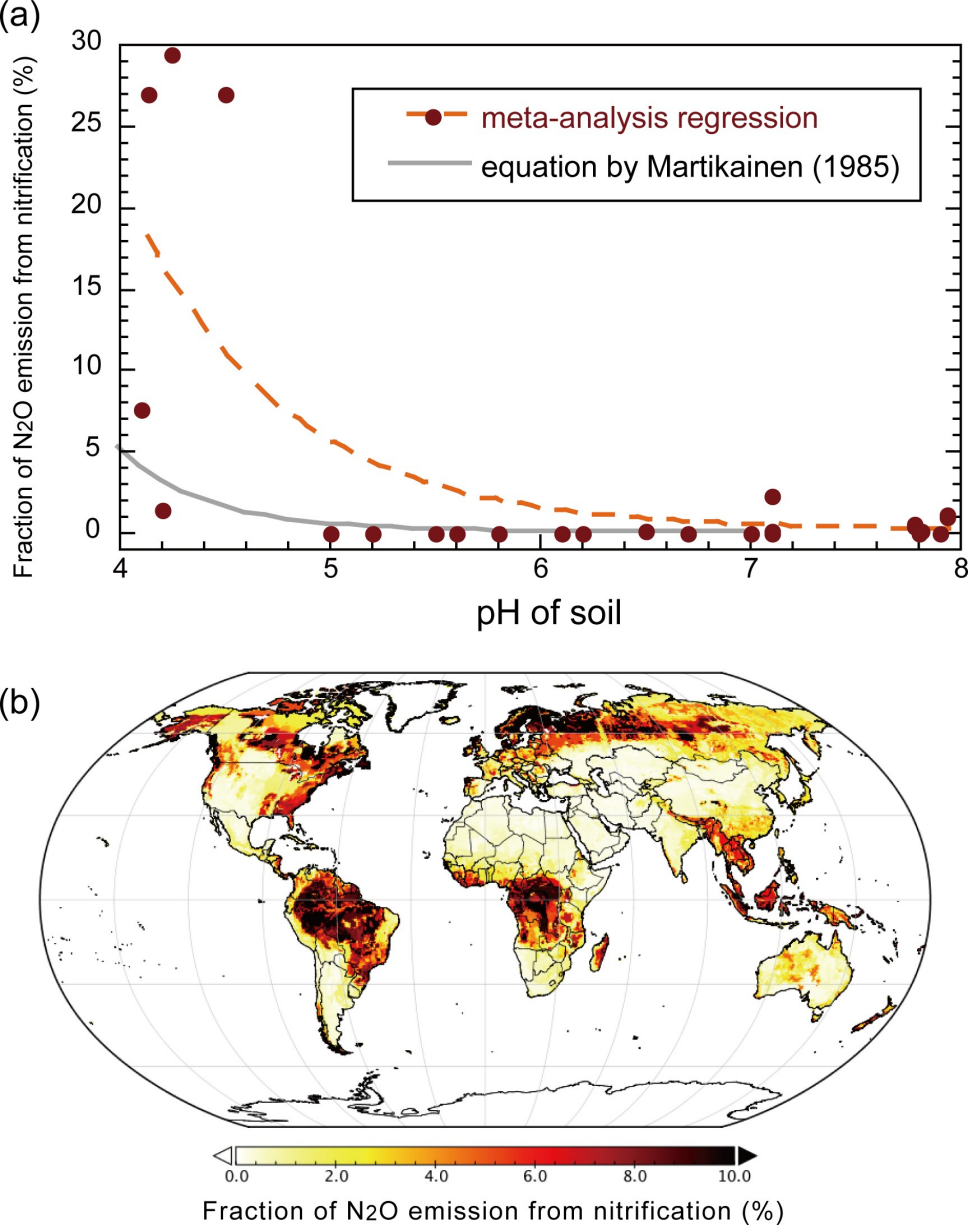
Parameterization of nitrification-associated N_2_O emission fraction, fN_2_O_nit_ as a function of soil pH. (a) Relationships in the meta-analysis data. Orange dashed curve is obtained by Gauss-Newton non-linear regression of an exponential function: fN_2_O_nit_ = 47.59 exp(–1.345 · pH) (*R*^2^ = 0.557). Grey curve shows an empirical function by Martikainen (1985) for reference. (b) Global distribution of fN_2_O_nit_ estimated using the regression curve and soil pH map (S1 Fig).

**Fig 8:**
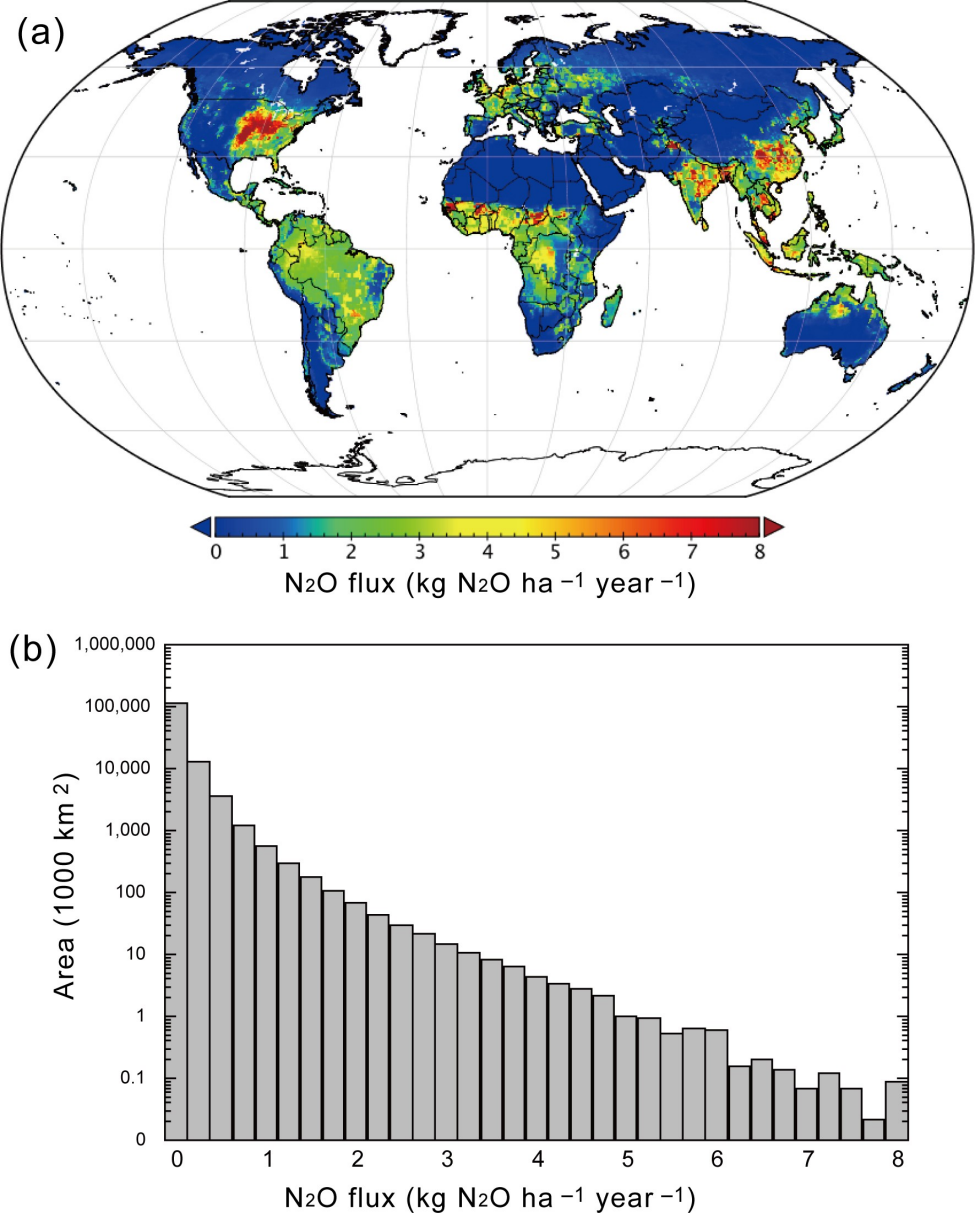
Simulated distribution of N_2_O emission using VISIT model with the pH-based fN_2_O_nit_ parameterization. (a) Global map for the 2000s and (b) frequency distribution of N_2_O emission intensity; note the log scale of y-axis.

In 54 records of the meta-analysis data, soil pH condition was included, allowing us to relate with fN_2_O_nit_ (Fig 7A). It was found that fN_2_O_nit_ takes higher values at acidic soil conditions with pH below 5 and lower values under neutral to alkaline soil conditions. We obtained a regression curve using exponential function, which gives slightly higher fN_2_O_nit_ values in comparison with the equation of Martikainen (1985) [40]. Using the soil pH map and the regression curve, global distribution of pH-based fN_2_O_nit_ was obtained (Fig 7B). As expected from the pH pattern, boreal conifer forest soils and humid tropical soils show higher fN_2_O_nit_ values. High fN_2_O_nit_ in humid tropics estimated by the present study is consistent with those by DNDC and DLEM parameterizations, while the three maps differ largely in temperate regions. When using the pH-based parameterization, total N_2_O emission in the 2000s was estimated as 16.8 Tg N_2_O yr^–1^ (25.2% by nitrification and 74.8% by denitrification). Global distribution of soil N_2_O emission was reasonably simulated (Fig 8; see S2 Fig for seasonal change), in comparison with those obtained by atmospheric inversion studies [56]. For example, high emissions from temperate croplands and tropical forests were well captured.

### Impacts on global N_2_O budget

**Fig 9:**
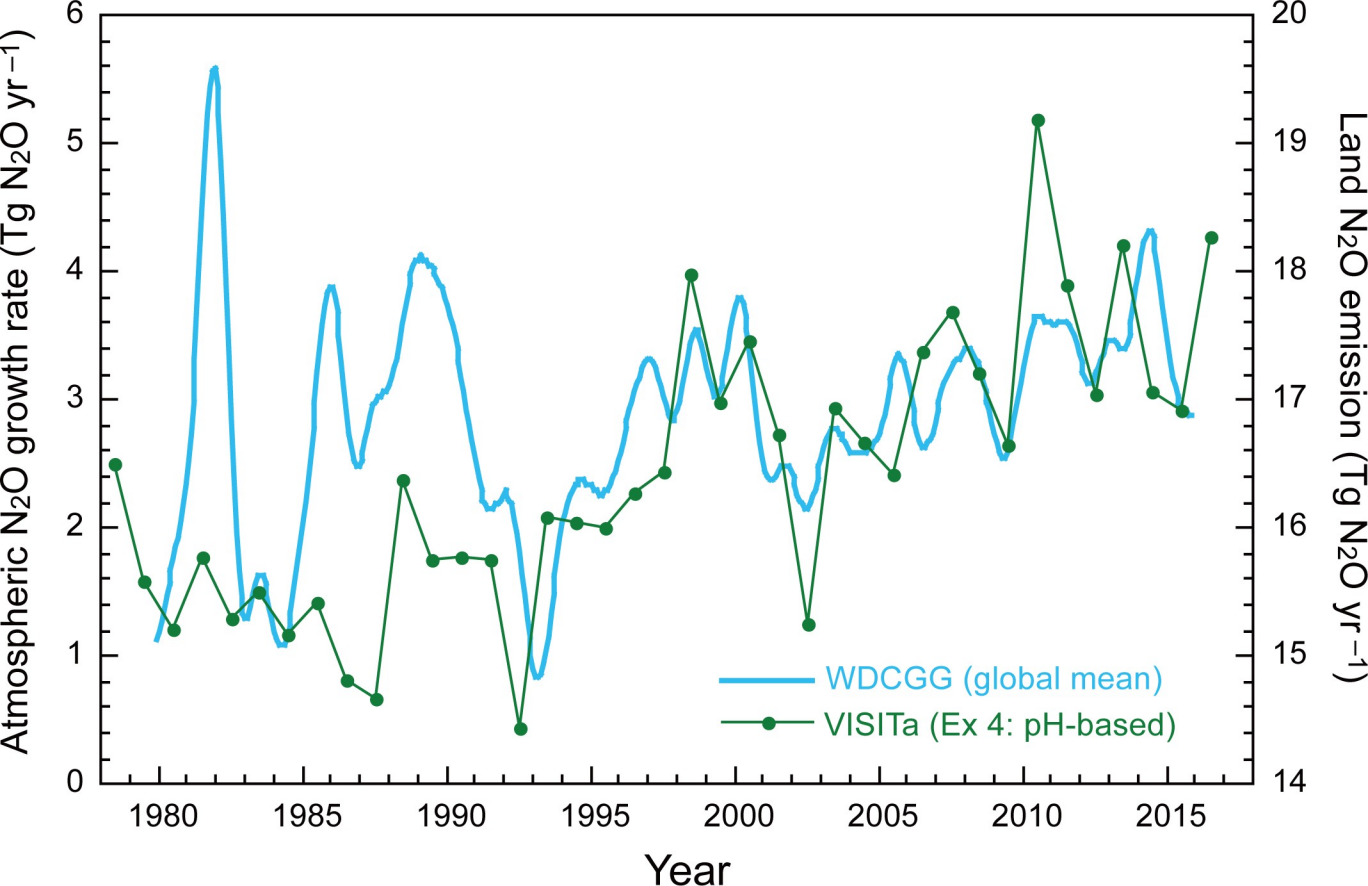
Time-series of total soil N_2_O emissions estimated using VISIT model with the pH-based fN_2_O_nit_ parameterization. Observed global mean growth rate of atmospheric N_2_O by the World Data Center for Greenhouse Gases (https://ds.data.jma.go.jp/gmd/wdcgg/wdcgg.html) are shown for reference.

**Fig 10:**
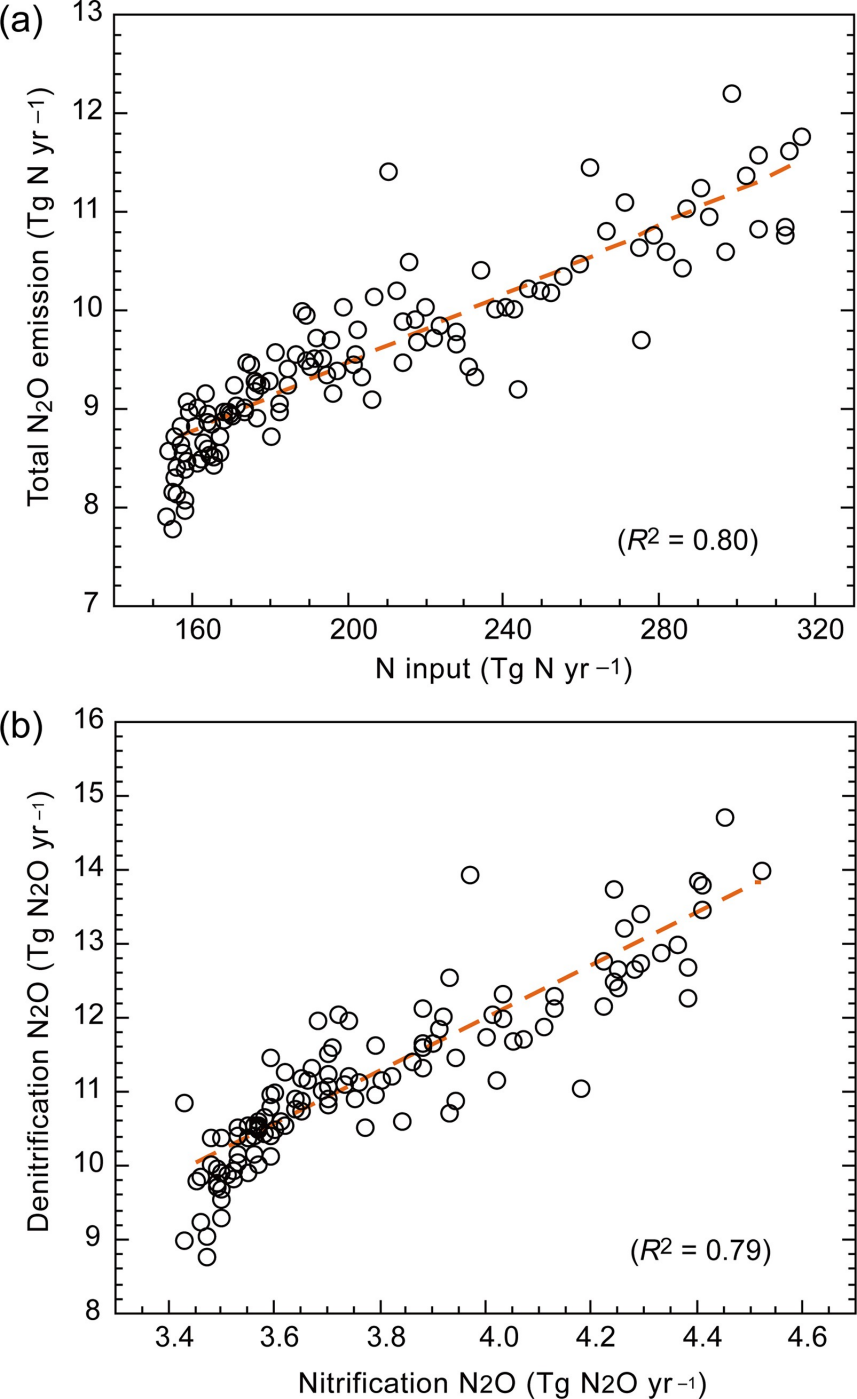
Relationships in the simulated global nitrogen budget by VISIT model with the pH-based parameterization of fN_2_O_nit_. (a) Total N_2_O emission related to nitrogen input by biological fixation, atmospheric deposition, and fertilizer. Orange dashed line shows liner regression: N_2_O emission = 0.0175 N-input + 5.94 (*R*^2^ = 0.804). (b) Relationship between nitrification- and denitrification-associated N_2_O emission: N_2_O-denitrification = 3.57 N_2_O-nitrification– 2.33 (*R*^2^ = 0.794). Simulation data from 1901 to 2015 were used.

The simulated N_2_O emission account for important features of the global budget. For example, interannual variability in the total N_2_O emission was comparable with the atmospheric growth rate especially after 1990, i.e. the period when ample observational data became available (Fig 9). The decline after the Mt. Pinatubo eruption in 1991 and following increase were well captured, implying the major impact of soil emission on the atmospheric N_2_O variability in recent decades. As clearly shown in the relationship between nitrogen input and N_2_O emission (Fig 10A), the historical increase of N_2_O emission in recent decades is mainly attributable to increased land N inputs by atmospheric deposition and fertilizer use. The slope, so-called emission factor, was estimated as 1.75%. This is a bit higher than the typical emission factor value of IPCC guideline [57], 1%, but note that the present estimate includes the effects of climate and land-use changes. In the model simulation, N_2_O emissions from nitrification and denitrification increased in parallel, as shown by the linear relationship between the two emissions (Fig 10B). Validating the N_2_O production scheme at broad scales is difficult even by comparing with inversion studies. In forthcoming studies, appropriate observations of N_2_O isotopomers may provide supporting evidences [58, 59].

Our meta-analysis and model simulations suggest the importance and uncertainty of fN_2_O_nit_ values in the evaluation of global N_2_O budget. Variability in a single parameter could cause a difference in total N_2_O emission of as much as 2.5 Tg N_2_O yr^–1^ (in the 2000s, 15.0 to 17.5 Tg N_2_O yr^–1^)—equivalent to the variability of 0.2 Pg CO_2_-C yr^–1^ (based on a global warming potential of 298 for N_2_O with 100-yr horizon [1]). The magnitude of the estimated N_2_O emission increase from the beginning to the end of the simulation was about 3.9 Tg N_2_O yr^–1^ in the simulations with different fN_2_O_nit_ values (comparable with a simulation by O-CN model [60] and a global synthesis [55]). Because the global nitrogen cycle would be further perturbed by human activities and climate change [5], the uncertainty in the present models can be a critical limiting factor for environmental management. Although global N_2_O budget may be constrained by using atmospheric observational data to some extent, in-depth understanding of flow components and their environmental regulations is essential to conduct effective nitrogen and climate managements.

## Concluding remarks

To our knowledge, this is the first study to have focused on fN_2_O_nit_ in a comprehensive manner. We should pay attention to the fact that this study used a limited number of observational data and extrapolated them to the global scale. Nevertheless, the dataset covering a variety of ecosystems and soils and the process-based model assessment gave us clues to better understanding of N_2_O cycle. Our findings gives an explanation for the results of the N_2_O model intercomparison project, which shows 20% of global soil N_2_O emission difference among terrestrial models [61]. In our analysis, selection of fN_2_O_nit_ affected the estimation of global N_2_O emission by about 15% (2.5/16 Tg N_2_O yr^–1^). Because of the scarcity of reliable observational data, our meta-analysis did not give a conclusive value or equation for fN_2_O_nit_. Most of observed values were low (<1%) but neglecting high values may result in underestimation of nitrification-associated N_2_O emission at broad scales. We found a potential and representative range of fN_2_O_nit_ values and a useful pH-based empirical model covering both natural and agricultural soils. This result should encourage extensive observations of nitrification-associated N_2_O emission and fN_2_O_nit_ by using a standardized protocol especially in the field. Although the present meta-analysis showed that the majority of empirical data were obtained by the C_2_H_2_ inhibition or isotopic tracer methods, further discussions on effective measurement strategy (e.g., spatial and temporal coverage and representativeness) are required for field and model researchers to improve model accuracy. Because of extreme complexity of the soil biogeochemical processes, it is inevitable to use simplified schemes like the ‘hole-in-a-pipe’ concept and bulk parameters like fN_2_O_nit_ to conduct simulations at broad scales. Because N_2_O has a high global warming potential, a small difference in estimated N_2_O emission can considerably influence the total greenhouse gas budget, as shown by our sensitivity simulations. To develop a better parameterization of fN_2_O_nit_ and other, related properties, further observations—especially in the field—and process studies of the nitrogen cycle and greenhouse gas emissions are critically important.

## Supporting information

S1 Table PRISMA 2009 check list. (DOC)

S2 Table List of search terms. (XLSX)

S3 Table Data extracted from the literature and used by the meta-analysis. (XLSX)

S1 Fig Map of soil pH (Global Soil Data Task, International Geosphere-Biosphere Program). (TIF)

S2 Fig Seasonal change in total N_2_O emission simulated by the VISIT model. (a) Norther winter (DJF: December, January, and February), (b) northern spring (MAM: March, April, and May), (c) northern summer (JJA: June, July, and August), and (d) northern autumn (SON: September, October, and November).
(TIF)
