# Supplementary figures and images for: Fraction of nitrous oxide production in nitrification and its effect on total soil emission: A meta-analysis and global-scale sensitivity analysis using a process-based model
Source: PLoS One. 2019 Jul 10;14(7):e0219159. doi: 10.1371/journal.pone.0219159 (PMC6619742; doi:10.1371/journal.pone.0219159)

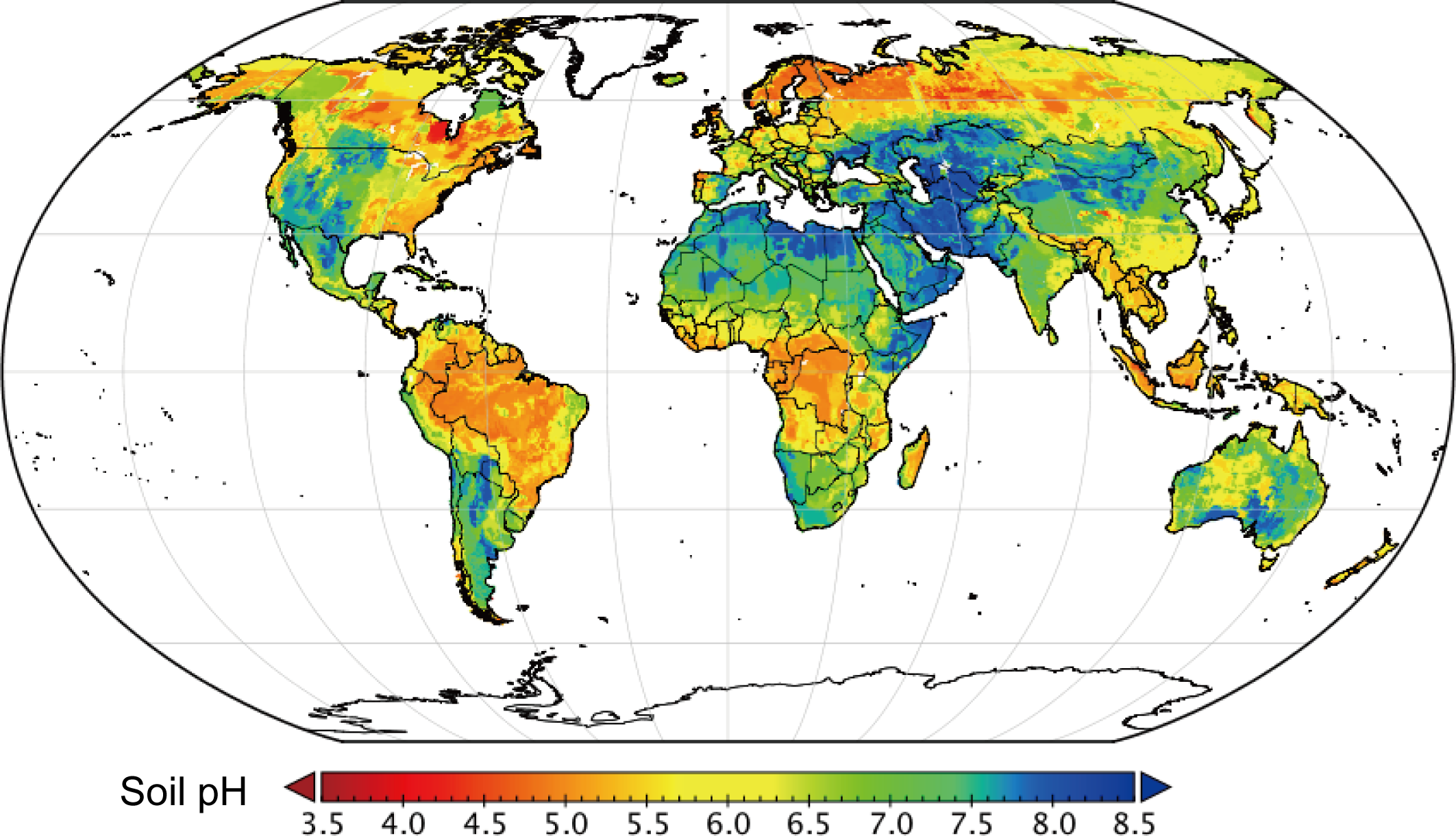

Supplement: S1 Fig — (TIF) [file pone.0219159.s004.tif]

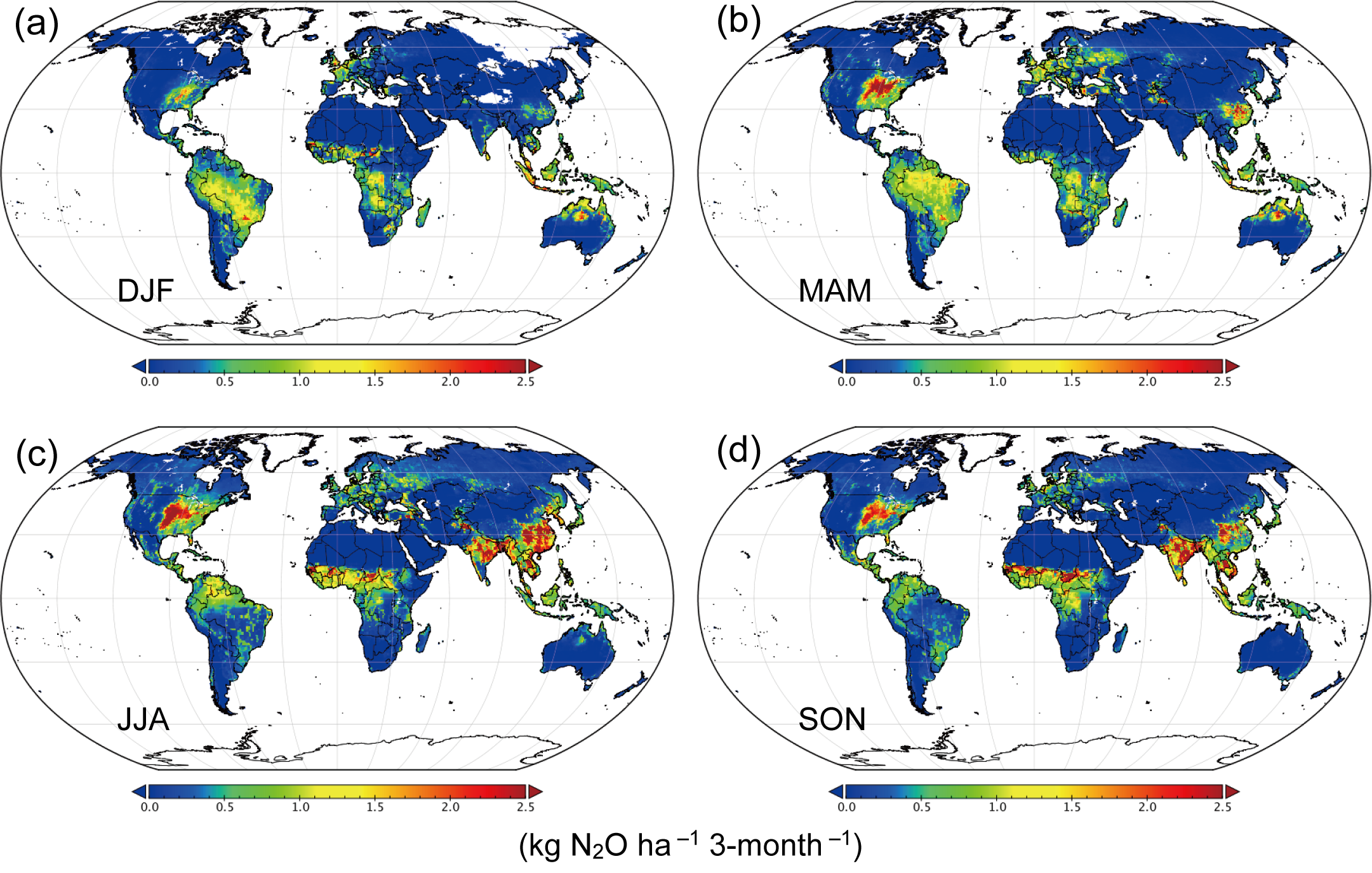

Supplement: S2 Fig — (a) Norther winter (DJF: December, January, and February), (b) northern spring (MAM: March, April, and May), (c) northern summer (JJA: June, July, and August), and (d) northern autumn (SON: September, October, and November). (TIF) [file pone.0219159.s005.tif]
